# Concurrent genomic assessment of circulating tumour cells and ctDNA to guide therapy in metastatic breast cancer

**DOI:** 10.1186/s12885-025-15187-5

**Published:** 2025-12-05

**Authors:** Rebecca C. Allsopp, Karen Page, Evie Wren, Georgios Nteliopoulos, Kelly L. T. Gleason, Gurdeep Matharu Lall, Shradha Bhagani, Emmanuel Acheampong, Marc K. Wadsley, Raoul Charles Coombes, Jacqueline A. Shaw

**Affiliations:** 1 Leicester Cancer Research Centre, School of Medical Sciences, University of Leicester, Robert Kilpatrick Clinical Sciences Building, Leicester Royal Infirmary, Leicester, LE2 7LX UK; 2 Department of Surgery and Cancer, Imperial College London, Hammersmith Hospital, Du Cane Road, London, W12 0NN UK; 3 The Breast Cancer Now Toby Robins Research Center, The Institute of Cancer Research, 237 Fulham Road, London, SW3 6JB UK

**Keywords:** Liquid biopsy, CTCs, CfDNA, WGA, SWGS, EpCAM, Metastatic breast cancer

## Abstract

**Background:**

Molecular analysis of actionable driver mutations and somatic copy number alterations (sCNAs) in circulating tumour DNA (ctDNA) and circulating tumour cells (CTCs) is increasingly being used to guide personalised medicine in patients with cancer. In previous CTC studies, high numbers of CTCs were needed for successful recovery of individual CTCs for molecular analysis at a time when patients typically have very short survival, limiting clinical applicability. Here, by performing longitudinal analyses of ctDNA and CTCs across a broad range of CTC counts, we hypothesized that CTCs could reveal synergistic and additional genomic information to ctDNA at points when therapeutic interventions could be considered in the follow up of patients with metastatic breast cancer (MBC).

**Methods:**

Eight patients underwent serial blood sampling. CTCs were captured via CellSearch-DEPArray from 7.5 mL (CellSave tubes), while 15 mL (EDTA tubes) was used for cfDNA extraction. A total of 58 cfDNA samples and 192 CTCs from the 8 patients were compared by shallow whole genome sequencing sWGS and targeted next generation sequencing using custom designed mutation panels (cfDNA; dual barcoding Ion AmpliSeq HD technology (556 hotspots across 24 genes) and CTCs; SingleSeq-compatible AmpliSeq technology across 539 of the 556 (97%) hotspots).

**Results:**

The majority of patient samples showed complementary genomic information in CTCs and ctDNA from the same blood sample. However, genome changes were detected in CTCs from some blood samples that were ctDNA negative despite progression providing actionable information at times when ctDNA analysis was not informative. Across the CTCs and ctDNA, common regions of loss included chromosome 13q14 containing the *RB1* gene, detected in 3 of 4 patients receiving CDK4/6 inhibitors and amplification of 17q12 containing the *ERBB2* gene in 2 of the 7 patients with HER2 negative metastatic disease, suggesting evolution to HER2 positive disease.

**Conclusions:**

Our study shows that CTCs provide key information that would have been missed by ctDNA monitoring alone and extends CTC and cfDNA genomic profiling to patients with a broad range of CTC counts for blood-based monitoring of HER2 status and other clinically actionable targets for informing treatment decisions in metastatic disease.

**Supplementary Information:**

The online version contains supplementary material available at 10.1186/s12885-025-15187-5.

## Introduction

Breast cancer (BC) is the second leading cause of death for women worldwide, with the number of incidences rising [1]. Approximately 30% of women go on to develop metastatic breast cancer (MBC) with a 5-year survival rate of 28% [2]. Intratumor heterogeneity presents the biggest therapeutic challenge, leading to selective expansion of resistant clones, treatment failure and poor overall survival [3]. A tumour informed approach is the ultimate goal, to optimise treatment choice and sequence subsequent lines of therapy to improve patient outcomes and quality of life.

Unlike solid tumour biopsy, liquid biopsy analysis of circulating tumour DNA (ctDNA) and circulating tumour cells (CTCs) has potential to capture the genomic complexity of the tumour and metastatic disease. For example, liquid biopsy can detect changes in human epidermal growth factor receptor 2 (HER2) [4, 5] and estrogen receptor (ER) status [6], which may differ between primary and metastatic lesions. Multiple studies have demonstrated the clinical utility of ctDNA to targeted therapy in MBC [7–9] as well as the efficacy of treatment decisions based on the total CTC count [10–12] and extracellular vesicles [13]. Building on a small number of multi-analyte exploratory studies [14–20] we hypothesise that CTCs provide complementary or additional actionable genomic information to ctDNA. By longitudinally sampling patients over a median 483 days (range 64—721), we concurrently assessed somatic copy number alterations (sCNAs) and driver mutations in individual CTCs, alongside matched cfDNA. 

Here, we focussed here on the longitudinal follow up of patients with a dynamic range of CTC counts (median 35, range 1—2,347) during treatment. We describe a workflow for concurrent analysis of sCNAs and driver mutations in individual CTCs and demonstrate its application for comprehensive genomic profiling of single CTCs in patients with metastatic disease at a time when molecular guided changes to treatment could be offered. We compared individual CTCs to matched cfDNA in the same patients and samples concluding that CTCs and cfDNA provide synergistic information to guide treatment choice through longitudinal sampling at time points spanning disease progression.

## Material and methods

### Patients and sample collection

Eight patients receiving treatment for MBC were recruited to the study and blood collections were aligned with their hospital visits through treatment. All gave written informed consent prior to participation and were over 18 years of age. The study protocol was approved by the Riverside Research Ethics Committee (Imperial College Healthcare NHS Trust), Tissue Bank Ethics/REC reference 22/WA/0214 and was conducted in accordance with Good Clinical Practice Guidelines and the Declaration of Helsinki. 7.5 mL blood was taken into CellSave preservative tubes for CellSearch® CTC capture and enumeration as described previously [14]. DEPArray™ recovered CTCs and white blood cells (WBCs; germline controls) were reduced to a volume of ~ 2 ul (PBS) and stored at −20 °C until WGA. CTC selection for whole genome amplification (WGA) was based on staining (nucleated DAPI + cells, lacking CD45 and expressing CK-PE) and visual morphological review for healthy viable cells. A further 15 ml blood was taken into K2 EDTA tubes (BD Biosciences, San Jose, USA) and processed to plasma and buffy coat within 2 h of collection and cfDNA and germline DNA were isolated, as described previously [21]. The concentration and quality of recovery of cfDNA was checked using the Agilent cfDNA ScreenTape Assay on the 4200 Bioanalyzer (Agilent Technologies, Santa Clara, USA). FFPE tumour DNA was extracted from 1 mm tumour tissue cores using the Qiagen GeneRead Kit (QIAGEN, Hilden, Germany) according to manufacturer’s instructions.

### WGA of template DNA

sWGS sCNA analysis of 300 pg cfDNA and FFPE tumour biopsy DNA (where available) was performed as described previously [22] for comparison and to monitor disease evolution over time. For single cells (CTCs and WBCs) where concurrent CN and mutation analysis was performed, the number of WGA cycles was increased from 12 to 15 to compensate for removal of 20% (3 ul) WGA product as template for custom assay mutation analysis. Pooled libraries were run on Ion 520™ chips (550 flows) and analysed as described previously [22]. The resulting reads were aligned to the hg19 reference genome using Ion Torrent™ Suite Software. Quality control (QC) metrics (defined in the manufacturer’s instructions) required sequencing data to exceed > 100,000 productive reads with a median absolute pairwise difference (MAPD) < 0.3. cfDNA sWGS analysis was performed as described previously [22]. Subsequent analysis using ichorCNA (https://github.com/broadinstitute/ichorCNA) (default parameters, 2 MB tile size, mapping quality threshold 15) was used to estimate the tumour fraction (TFx) and determine the regions of CN change [23]. Actionable amplification and deletion analysis was performed using Bedtools (v2.31.0) to intersect copy number variations with OncoKB Actionable Genes (https://www.oncokb.org/actionable-genes#sections=Tx). Copy number states were classified as heterozygous deletion (HETD, 1 copy), copy neutral (NEUT, 2 copies), copy gain (GAIN, 3 copies), amplification (AMP, 4 copies) and high-level amplification (HLAMP, 5 + copies) [23].

### Mutation analysis

Targeted custom sequencing panels for both cfDNA and single CTCs were designed using the Ion AmpliSeq designer tool (v7.42 and v7.68 respectively). cfDNA and matched leukocyte control to identify and exclude potential Chip mutations, utilised dual barcoding Ion AmpliSeq HD technology (Thermo Fisher Scientific) covering 556 hotspots in 87 amplicons, across 24 genes. The analytical sensitivity of the assay is ~ 0.1% VAF (using 30 ng DNA template according to the manufacturer’s protocol). However, the true limit of detection is both locus and sample dependent; reported for each variant by the AmpliSeq™ HD caller and detailed in the supplementary material. CTCs and WBC germline controls were analysed using SingleSeq-compatible AmpliSeq technology, covering 539 of 556 (97%) hotspots (Supp Table 1). Libraries were amplified according to the manufacturer’s protocol, comprising 21 PCR cycles plus 7 equaliser cycles. Variant detection (using the AmpliSeq variant caller) in single cells is binary at each locus (absent, heterozygous or homozygous). Apparent absence of a variant may result from allelic dropout, PCR bias or insufficient coverage rather than true absence, and so non-detection compared with matched cfDNA was interpreted in this context. Resulting libraries were screened using the Agilent HSD1000 DNA ScreenTape Assay on the 4200 Bioanalyzer. Sixteen cfDNA libraries and twelve single-cell libraries each diluted to 100 pM were pooled respectively and sequenced on 540 chips using the Ion S5 system. Reads were aligned and analysed with default settings. Variants were filtered with mapping quality > 15 and quality by depth > 2. All COSMIC-associated variants were manually reviewed in IGV (v2.3.82).

## Results

Eight patients with radiologically-confirmed MBC were serially sampled through treatment spanning a median of 483 days (range 64 to 721) (Supp Table 2). All patients had a diagnosis of ER-positive and HER2-negative primary BC, and all except for patient 3 remained HER2-negative upon metastatic relapse (Table 1). Patient 2 had consistently high CTC counts across all time points ranging from 390 to 2,347 (median of 1,140). The other patients had a lower median CTC count (median 26, range 1–714), peaking at points of disease progression (Supp Table 2). Although based on small numbers, correlation analysis revealed strong correlation between total plasma cfDNA concentration and CTC count (rho 0.6, *p* < 0.05) and a moderate correlation between either the cfDNA level or CTC count with ALP (rho 0.47 and 0.52 respectively *p* < 0.05) (Supp Table 2) consistent with previous findings [24] (Supp Table 2).

**Table 1:** Estrogen receptor (ER), progesterone receptor (PR) and HER2 receptor status at primary and metastatic biopsy

	**Primary tumour characteristics**		**Metastatic disease**	
**Patient ID**	**Grade and subtype**	**ER/PR/HER2 status**	**ER/PR/HER2 status**	**Duration of sampling (days)**
1	Unk	ER+ HER2-	ER+ PR+ HER2-	722
2	2 IDC	ER+ PR+ HER2-	ER+ PR- HER2-	382
3	2 IDC	ER+ PR- HER2-	ER- PR- HER2+	92
4	2 IDC	ER+ PR+ HER2-	ER+ PR+ HER2-	591
5	3 IDC	ER+ PR+ HER2-	ER+ PR+ HER2-	589
6	2 IDC	ER+ PR+ HER2-	ER+ HER2-	638
7	2 IDC	ER+ PR+ HER2-	ER+ PR+ HER2-	98
8	1 IDC	ER+ PR+ HER2-	Unk	96

*Unk* Information unknown

**Fig. 1:**
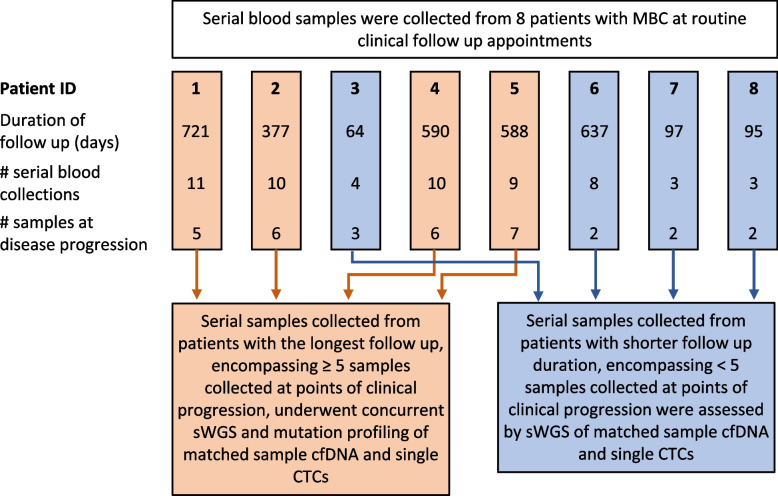
Study diagram showing patients and samples analysed

Across all patients, a total of 274 samples were identified for genomic profiling comprising 58 cfDNA samples (3 to 11 samples per patient), 192 single CTCs (median of 4 per time point collected across 33 sampling time points), 14 WBCs, 6 tumour FFPE samples (from 4 patients) and 4 buffy coat samples. All FFPE DNA, cfDNA samples and single cells were analysed by sWGS. Supp Fig. 1 shows a representative example of CellSearch®-defined, DEPArray-recovered single cells (CTCs and WBCs) from patient 5 that were visually selected and progressed for sWGS, highlighting individual cells that either achieved or failed to achieve sequencing QC thresholds. Focussing on the patients with ≥ 5 serial samples taken at points of radiological disease progression (Fig. 1, Supp Table 2), concurrent mutation analysis was performed. This included patients 1, 2, 4 and 5 with a combined 28 serial cfDNA samples, 30 CTCs and a small number of WBCs (where available) alongside buffy coat DNA samples. Details of the successfully DEPArray-routed, recovered and sequenced CTCs and WBCs per sample and patient are provided in Supp Table 3.

### sCNA analysis shows clonal copy number changes in paired plasma cfDNA and CTCs

Samples achieving QC parameters for sCNA analysis were 5/6 (83%) FFPE DNA samples, 56/58 (97%) cfDNA samples, 91/192 (47%) CTCs and 3/14 (27%) WBCs. Median productive reads and MAPD are summarised in Supp Table 4. The majority of CTCs analysed came from patients 1, 2 and 5 (30, 33 and 9 CTCs respectively). In contrast, more modest numbers of CTCs were available for patients 3, 4, 6, 7 and 8 that also achieved QC thresholds. Thirty of the 56 (54%) cfDNA samples had sCNAs detected (median 9 regions, range 2 to 17) with a median TFx of 0.35 (range 0.18 to 0.67), whereas 86/91 (95%) single CTCs had sCNAs detected across a median 11 regions (range 2 to 22) (Supp Table 5). The remaining 5 CellSearch® defined ‘CTCs’ each had flat sWGS profiles and cannot be confirmed as tumour derived cells by sWGS analysis.

**Fig. 2:**
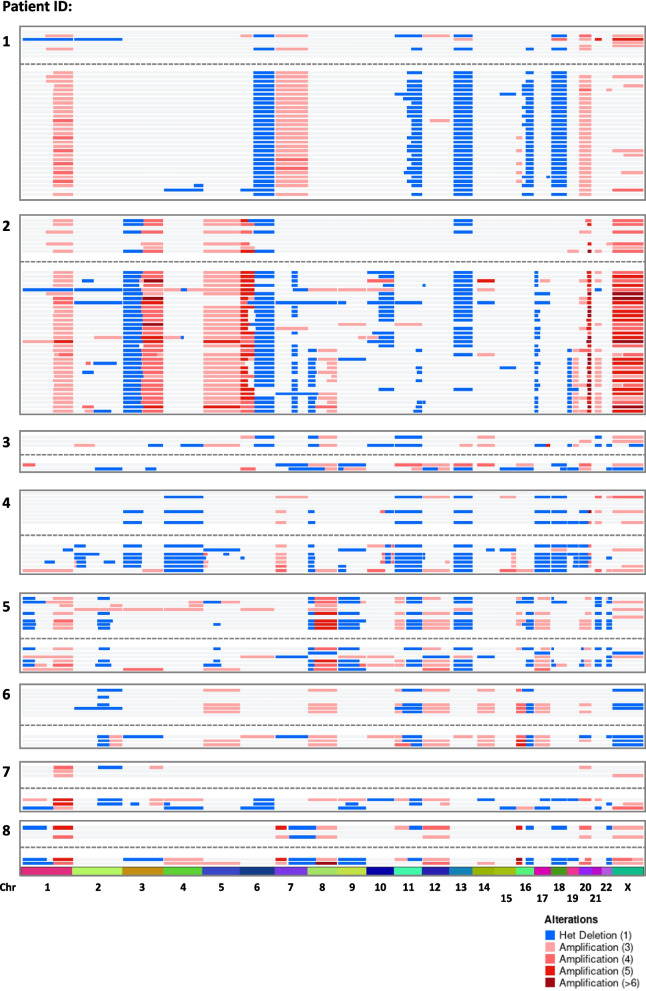
Oncoprint showing the sWGS CN changes within longitudinal MBC patient samples. For each patient, CN alterations detected in cfDNA are shown above those detected in matched single CTCs. cfDNA and CTC profiles are separated by a dotted line to indicate the different analytes

Individual patient sCNAs were highly conserved across longitudinal samples and CTCs generally had more regions of CN gain and loss than the matched cfDNA (Fig. 2). Examples of genes within commonly amplified chromosomal regions include *PIK3CA* (45/147 samples) and *FGFR1/2* (32/147 samples). Of significance all 8 patients had HER2 negative primary disease, one of whom (patient 3) had a HER2-positive metastatic biopsy (Table 1) detected in cfDNA and one CTC but below QC thresholds. Gain or amplification of the region containing *ERBB2* was detected within cfDNA samples and CTCs spanning multiple sampling points in 2 other patients (5 and 6) and in a single CTC from patient 2 during progression (discussed in more detail below). Common regions of loss across the 8 patients contained genes *BRCA1* and/or *BRCA2* (78/147 samples), *RB1* (73/147 samples) and *BAP1* (46/147 samples) (Supp Table 5).

### Mutation analysis shows common and unique mutations in cfDNA and CTCs

Of the 28 cfDNA samples and buffy coat controls analysed using a highly sensitive Ampliseq HD assay, all samples achieved minimum QC sequencing requirements (Supp Table 6). 23/28 (82%) cfDNA samples had somatic mutations detected with an associated COSMIC ID (Supp Table 7). Of the single cells, 29/30 CTCs (97%) and 3/3 WBCs (100%) achieved desired mutation sequencing metrics (Supp Table 8) with 23/29 (79%) of CTCs containing mutations with an associated COSMIC ID (Supp Table 9) in addition to 6 with either germline or intronic variant calls only. In all cases, CTCs confirmed the matched cfDNA identified driver mutations (including *ESR1 E380Q, GATA3 R306Q, PIK3CA H1047R, PIK3CA M1043V, TP53 H179Q, TP53 G245V* and RB1 Q217*)*.* Outside of driver mutations, other variants were identified that were unique to cfDNA (6/13) and to CTCs (7/14) respectively. Germline SNPs detected in matched cfDNA and BC were also confirmed within WBC analysis confirming sample identity (Supp Table 7 and 9).

### Patient-by-patient breakdown of genomic alterations

Table 2 presents a summary of sWGS and driver mutation analyses, concordance between cfDNA and CTCs and the associated clinical significance.

**Table 2:**
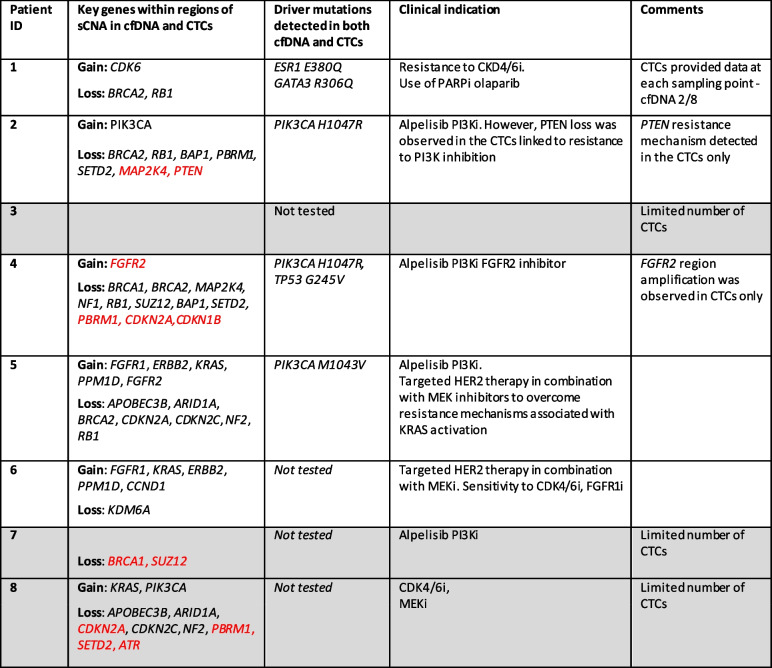
Key sWGS-sCNA and hotspot driver mutations detected in more than one serial sample. Findings highlighted in red text were detected solely within CTCs and not matched cfDNA. Clinical implications, potential therapeutic strategy and additional comments are also indicated. Three patients lacked sufficient number of samples for comprehensive analysis (indicated in grey)

**Fig. 3:**
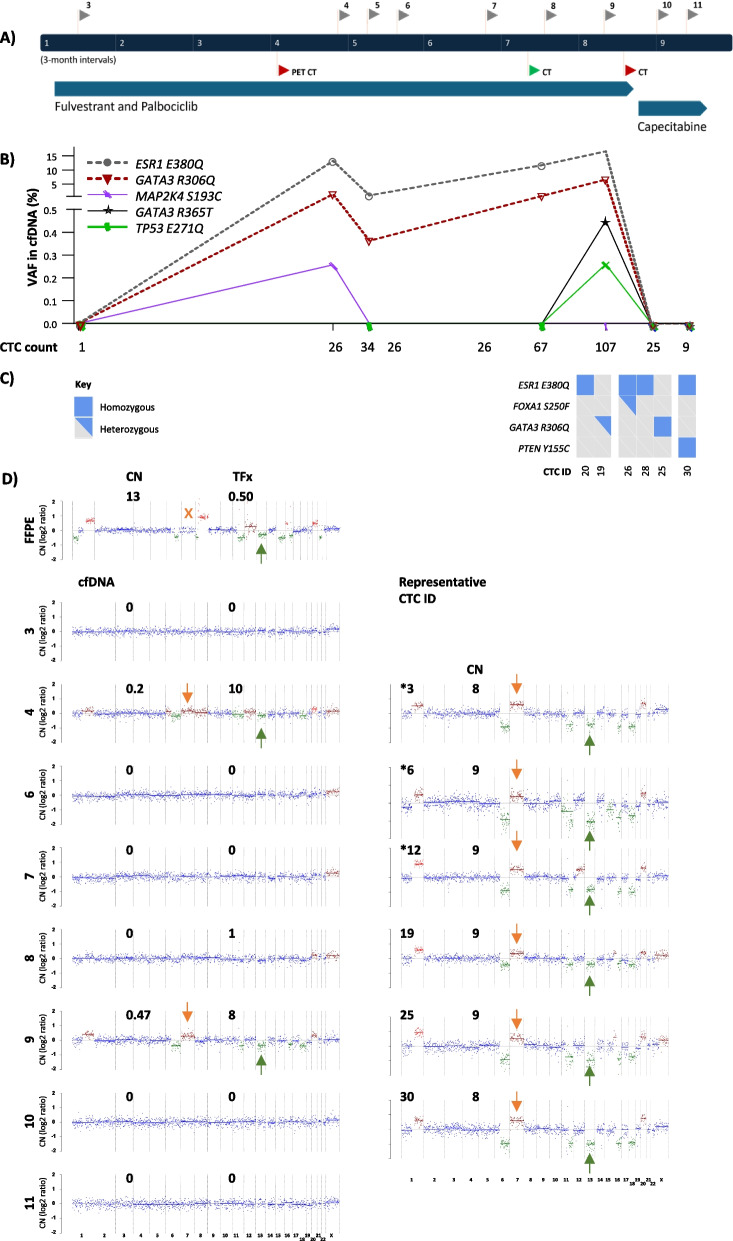
Patient 1 longitudinal sCNA and mutation analysis in CTCs and cfDNA. **A** Treatment and radiological response timeline whereby cfDNA sample ID is highlighted on the top of the timeline and green and red flags below the timeline indicate mixed response and disease progression respectively. **B** Mutation analysis of cfDNA (samples 3–5 & 8–11) with CTC counts at each time point on the x-axis. Dotted lines indicate mutations concurrently detected in CTCs. **C** Matched single CTCs at each sampling point, illustrating homogeneous or heterogeneous mutation expression. **D** sWGS profiles showing loss of the region containing BRCA2 and RB1 (green arrow) in relapse FFPE DNA, 24/30 CTCs across 5/6 sampling points, and 2/8 cfDNA samples. Amplification of CDK6 (orange arrow) is observed in 29/30 CTCs across all 6 sampling points and 2/10 cfDNA samples

*Patient 1* Plasma cfDNA mutation analysis revealed *ESR1 E380Q* and *GATA3 R306Q* mutations in 4/8 cfDNA samples tested. The % VAF increased (0.93 to 16.6 and 0.36 to 6.61 for E380Q and R306Q respectively) during radiologically informed stable disease whilst on treatment with fulvestrant and palbociclib and then resolved upon switch to capecitabine (Fig. 3A + B, Supp Table 7). CTC analysis also detected the *ESR1 E380Q* (homozygous) and *GATA3 R306Q* (homozygous at later sampling point) mutations in distinct CTCs, suggesting different sub-clonal origins (Fig. 3C, Supp Table 9). Additional low % VAF mutations (< 0.5%) (*MAP2K4 S193C, GATA3 R365T* and *TP53 E271Q)* were detected at single plasma sampling points (cfDNA 3, 9 and 3 respectively; Supp Table 7) and were undetected in CTCs, whereas the *FOXA1 S250F* and *PTEN Y155C* mutations were detected exclusively in individual CTCs at late sampling time points (Fig. 3, Supp Table 9).

sWGS showed deletion of chromosomal regions containing *BRCA2* and *RB1* genes in her local relapse biopsy FFPE DNA (Supp Table 5, Fig. 3D). Consistent loss of the region containing *RB1* and gain of the region containing *CDK6* were captured in 2/8 cfDNA sampling points and 29/29 CTCs spanning 6/6 longitudinal sampling points whilst she was receiving fulvestrant and palbociclib. The same samples also demonstrating loss of the region containing *BRCA2*. Finally, amplification of the region of chromosome 8 containing the *FGFR1* gene detected in the primary tumour DNA was subsequently detected in a single early cfDNA but not in any CTCs (Supp Table 5, Fig. 3D).

**Fig. 4:**
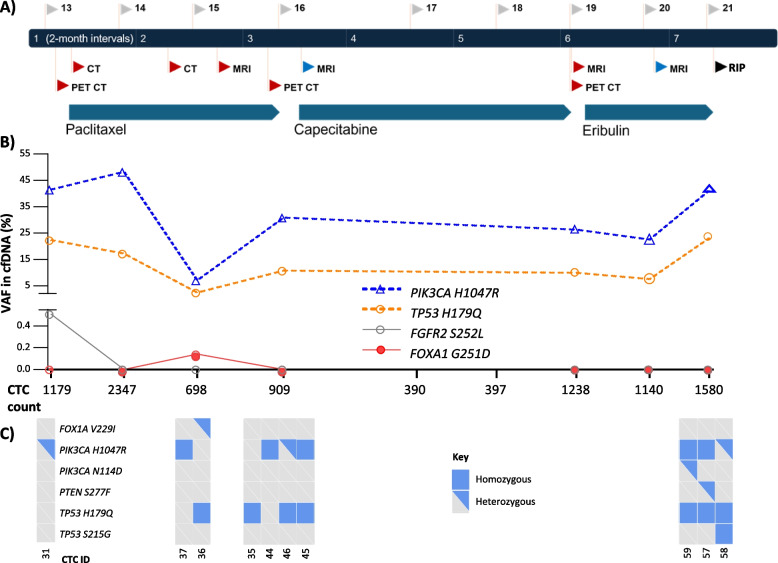
Patient 2 longitudinal mutation analysis in CTCs and cfDNA.** A** Treatment and radiological response timeline whereby cfDNA sample ID is highlighted on the top of the timeline and blue and red flags below the timeline indicate stable disease and disease progression respectively. **B** Mutation analysis of cfDNA (samples 13–16 + 19–21; 17 + 18 not tested) with CTC counts at each time point on the x-axis. Dotted lines indicate mutations concurrently detected in CTCs. **C** Matched single CTCs at each sampling point, illustrating homogeneous or heterogeneous mutation expression

*Patient 2* cfDNA mutation analysis detected the *PIK3CA* H1047R mutation at high % VAF (range 7.0 to 48.0%) and *TP53* H179Q mutation (range 2.4 to 23.0%) across all cfDNA sampling time points and CTCs (Fig. 4 A + B, Supp Table 7 + 9). These mutations persisted throughout treatment with paclitaxel, capecitabine and eribulin with the % VAF tracking with total cfDNA levels (Supp Table 2). Lower VAF *FGFR2* S252L (0.51%) and *FOXA1* G251H (0.14%) mutations were also detected in the cfDNA at single sampling points that were undetected in the CTCs investigated. In contrast, CTCs had *FOXA1* V229I*, PIK3CA* N114D, *PTEN* S227F and *TP53* S215G mutations, each detected in a single CTC that were not detected in cfDNA (Fig. 4C, Supp Table 7 + 9).

Consistent with *PIK3CA* gene mutation, sWGS revealed amplification of the region containing *PIK3CA* in the primary FFPE tissue DNA, 6/9 cfDNA samples and 33/33 CTCs across 7 sample time points (Supp Table 10). Of significance, 15/33 CTCs (spanning 4 sampling points) demonstrated loss of the region containing *PTEN* (Supp Table 10), undetected within the cfDNA (Supp Fig. 2). Regions of loss detected within cfDNA and CTCs contained *RB1* (detected at a time when the patient was receiving CDK4/6 inhibitor treatment), *BAP1* (4/8 cfDNA samples and 33/33 CTCs) and *BRCA2* (3/8 cfDNA samples and 19/33 CTCs spanning 5 time points) (Supp Tables 5 and 10). Additional information from the CTCs included amplification of the region containing *FGFR2* (in a single CTCs from the 1 st and 3rd timepoints), and amplification of *ERBB2* and *FGFR1* in single CTCs at distinct time points (Supp Table 10); all undetected within matched cfDNA (Supp Table 5).

*Patient 3* had 3 blood samples collected, however only 2 of 14 CTCs isolated passed sequencing QC. CN changes were detected in the first two plasma timepoints, when her disease was progressing on treatment. The third cfDNA sample had no CN changes detected despite disease progression, however the matched single CTC had 18 regions of CN change (Supp Fig. 3 and Supp Table 5). This patient also had HER2 positive metastatic biopsy evidence of *ERBB2* gain in a single CTC but below QC thresholds.

*Patient 4* Mutation analysis of serial cfDNA samples highlighted high VAF, *PIK3CA* H1047R (range 6.6 to 37.0%) and *TP53* G245V (range 2.4 to 40.2%) driver mutations, plus a lower VAF putative sub-clonal *RB1* Q217* mutation (range 0.25 to 3.0%) also detected in matched CTCs that persisted through changes in treatment (Supp Fig. 4 A + B). A low VAF *ERBB2* mutation (*I655V*, 0.21%) was also detected in cfDNA (cfDNA sample 27) but undetected in CTCs (Supp Tables 7 + 9).

sCNAs were detected in 3/9 serial cfDNAs, with gain in the region containing the *CDK6* gene and loss of regions including *BRCA1* and *BRCA2* (Supp Table 10), detected in 3/9 cfDNA samples and in 6/7 CTCs spanning 3 time points. Furthermore, amplification of the region containing *FGFR2* was detected in 4/7 CTCs from 2 time points, undetected within matched cfDNA (Supp Table 5 + 10, Supp Fig. 4 C). Finally, amplification of regions containing *FGFR1, PIK3CA* and *KRAS* were detected in a single CTC at the last sampling time point, undetected within cfDNA.

**Fig. 5:**
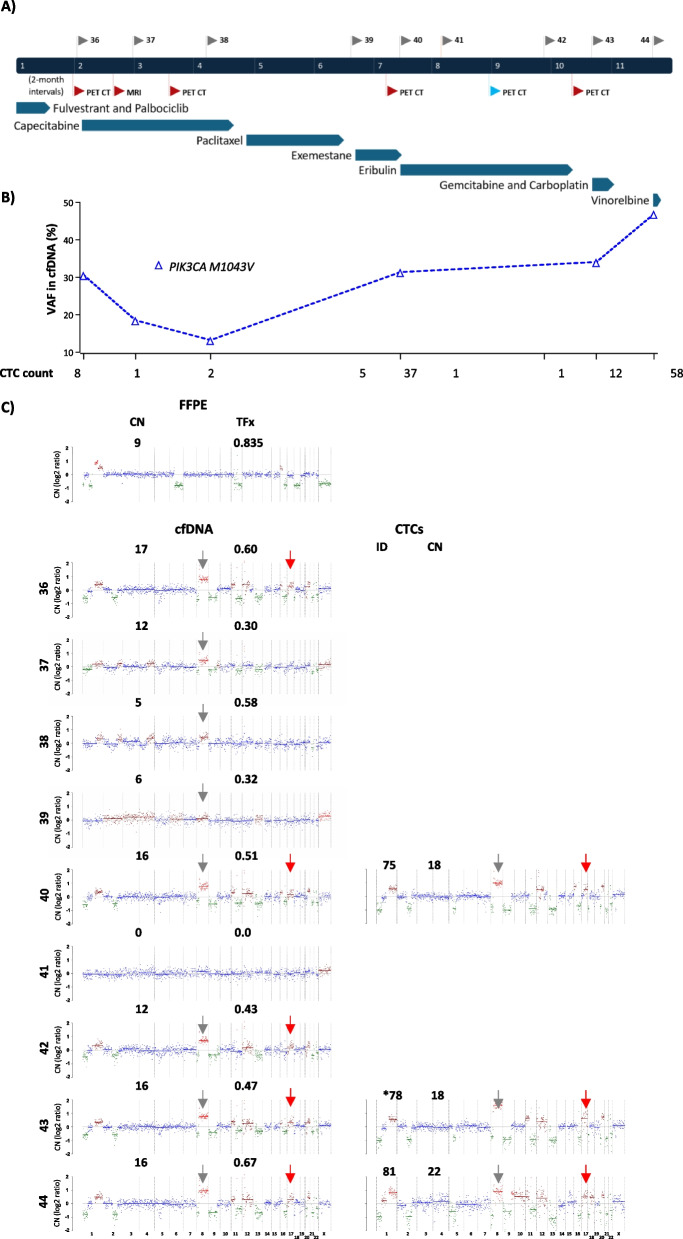
Patient 5 longitudinal sWGS-sCNA and mutation analysis in CTCs and cfDNA (**A**) Treatment and radiological response timeline, with cfDNA sample IDs shown above the timeline. Blue and red flags below indicate stable disease and disease progression, respectively. **B** Mutation analysis of cfDNA (samples 36, 37, 38, 40, 43 and 44) with CTC counts at each time point on the x-axis. The dotted line indicates that the mutation was concurrently detected in CTCs. **C** sWGS copy number profiles, showing amplification above the normal (blue) baseline highlighted in red. Red arrows indicate the region of chromosome 17 containing *ERBB2* and grey arrows indicate the region of chromosome 8 containing *FGFR1*

*Patient 5* Mutation analysis of patient 5 cfDNA samples revealed a consistent high % VAF *PIK3CA M1043V* mutation (range 13.6 to 47.2%), which declined upon capecitabine treatment before rising on all following treatments (Fig. 5A + B). This mutation was also detected in a single CTC from the final sampling point. Conversely, CTC analysis detected *CDH1 P245L* in the penultimate CTC sampling point, undetected in the matched cfDNA. Lastly, 2 low VAF putative Chip mutations (*TP53 G245D* and *TP53 Y220C*), were detected in the BC DNA (Supp Tables 7 + 9).

Of significance, patient 5 presented with HER2 negative primary and metastatic disease but gain of the region containing *ERBB2* was detected in 5/9 cfDNA samples and gain or amplifcation was detected in 5/6 CTCs spanning 3 time points (Supp Table 10). Further regions of amplification contained the genes *FGFR1* in 8/9 cfDNA samples and 5/7 CTCs spanning 3 time points and gain of *KRAS* in 5/9 cfDNA samples and 4/6 CTCs spanning 3 time points. Of note, none of these sCNAs were detected in the FFPE primary tumour tissue DNA (Supp Table 5, Fig. 5). For this patient, loss of the region containing *BRCA2* was detected in 6/9 cfDNAs and 5/6 CTCs spanning 3 time points along with loss of regions containing *APOBEC3B, ARID1A, CDKN2A, CDKN2C, NF2 and RB1* in cfDNA and CTCs (Supp Table 5).

*Patient 6* Analysis of cfDNA demonstrated amplification of the regions containing *ERBB2, FGFR1, KRAS* and *PPM1D* in 3 of 8 serial cfDNA samples (IDs 49–51) while on eribulin but with stable disease by CT. These were also detected within 3/3 single CTCs isolated from a single matched time point (Supp Fig. 5 and Supp Table 5).

*Patient 7* The 3 serial cfDNA samples analysed had few consistent CN changes detected apart from gain of chromosome 1q. Amplification of the region containing *PIK3CA* was also detected in the first cfDNA sample and one of 2 matched CTCs from this time point but was not detected in the 2 later samples (Supp Fig. 6 A + B).

*Patient 8* Amplification of the region containing *KRAS* was detected in the first and third cfDNA samples and in the 2/3 CTC from the first time point (Supp Fig. 6 C + D).

## Discussion

Given the invasive nature and difficulty in obtaining a tissue biopsy at relapse and progression, blood based liquid biopsy provides an attractive approach to monitor the cancer genome through treatment. Here, we demonstrate a workflow for the concurrent analysis of sCNAs and driver mutations in cfDNA and individual CellSearch® CTCs in the follow up of patients with MBC at a time when molecular guided changes to treatment could be offered.

Across the 8 patients, sWGS-sCNA analysis of single CTCs revealed largely clonal sCNA profiles for each patient, with some subclonal changes that evolved over time. The majority (86 of 91; 95%) of all single CTCs demonstrated genome wide sCNAs and actionable changes were detected from samples with a range of CTC counts (the lowest containing just ten CellSearch CTCs) at clinically relevant points. Some sCNAs detected within CTCs were undetected within the matched cfDNA, suggesting putative sub-clonal, treatment resistant alterations that are either too dilute to detect in plasma cfDNA or not present in the ctDNA fraction, supporting continuous evaluation of CTCs in patients with advanced BC [25]. Just 5 of the 91 successfully analysed CTCs showed flat copy number profiles despite meeting CellSearch CTC criteria, similarly reported in other studies [26].

In contrast, only 30 of 56 (54%) matched cfDNA samples had sCNAs detected. The variation in plasma cfDNA levels associated with disease progression and treatment response, coupled with mutational heterogeneity, accounting for some samples being ‘ctDNA negative’ or a non-informative result. Critically, in such situations, CTCs were able to provide the information when cfDNA could not. The regions of sCNA observed across cfDNA and CTCs herein were consistent with other MBC series [27, 28].

### Clinical implications

For patient 1, CTCs consistently recapitulated the sCNAs detected in the primary tumour FFPE DNA (amplification of the region containing CDK6 and deletion of the region containing RB1), that was undetected in the majority of cfDNA samples suggesting that CTCs may provide a more faithful reflection of tumour biology; in this case intrinsic resistance to CDK4/6 inhibitors from the time of primary surgery [29]. Furthermore, overexpression of CDK6 can decrease dependency on estrogen, undermining the role of fulvestrant [30]. Evidence from study NCT03007979 similarly demonstrated how sWGS assessment of ctDNA can provide a low-cost approach to monitor progression of HR +/HER2- MBC patients who are unlikely to respond to endocrine therapy combined with CDK4/6 inhibition [31]. Mutation analysis revealed the presence of the *ESR1 E380Q* gene mutation in all serial cfDNA samples, confirmed as homozygous by CTC analysis, associated with ligand-independent estrogen receptor activation and reduced sensitivity to fulvestrant [32]. Despite this biomarker evidence of likely resistance to ongoing fulvestrant and palbociclib, this patient remained stable for a prolonged period of time before radiological progression and a switch to capecitabine chemotherapy. Several trials aim to address the best course of treatment upon emergence of *ESR1* mutations. Based on the genomic information described above, trials would indicate the use of 2nd generation CDK4/6 inhibitors (SERENA NCT04964934), or the use of SERDs (OPERA-1 NCT06016738 and EMERALD NCT03778931) and novel combination of Samuraciclib (first in class selective oral CDK7 inhibitor [33]) with vepdegestrant a PROTAC® ER degrader, in women with ER +, HER2- MBC who have previously received a CDK4/6 inhibitor (TACTIVE-U NCT06125522).

This patient also demonstrated loss of the region containing BRCA2 in her primary and metastatic disease. Whilst BRCA1/2 mutations are a well-established biomarker of sensitivity to PARPi such as olaparib [34] emerging evidence also supports its use in tumours with HRD resulting from somatic copy number losses [35]. Furthermore, the *GATA3 R306Q* mutation detected in all ctDNA and CTCs samples is also linked to poor response to hormonal therapy and poor prognosis [36]. Whilst *GATA3* mutations are not yet targetable, synthetic lethal interactions between GATA3 and MDM2 in ER + BC supports pharmacological inhibition of MDM2, shown to significantly impair tumour growth in *GATA3*-deficient models [36].

For patient 2, sWGS-sCNA profiling of both cfDNA and CTCs revealed amplification of the region containing *PIK3CA*, with targeted sequencing confirming a persistently high VAF *PIK3CA* H1047R mutation alongside *TP53* H179Q, likely contributing to resistance to prior endocrine therapy [37] and associated with poor prognosis and chemotherapy resistance [38, 39]. Furthermore, persistently elevated CTC counts across all time points also correlate with inferior treatment response and survival [40]. *PIK3CA* CN gain is reported to occur preferentially in *PIK3CA* mutant tumours potentially leading to more aggressive disease and a different response to treatment with supporting the hypothesis of a potential additive effect of mutations and gain to oncogenesis. Treatment decisions currently rely solely on mutation status, but studies indicate that CN may help refine the prognostic and predictive role of mutations [41]. Further validation is needed before integration into clinical decision making. Of importance, loss of the region containing *PTEN* was detected in multiple CTCs across serial collections but not in matched cfDNA. This observation is relevant as clinical trials such as BELLE [42, 43] and SOLAR1 established PI3K inhibitors like Alpelisib as effective therapies for PIK3CA-mutant BC, with regulatory approval in combination with fulvestrant [7] or nab-paclitaxel [44]. However, *PTEN* loss has been linked to resistance to PI3K inhibition [45] raising questions about the durability of such strategies in this patient.

Beyond these events, CTC profiling also identified subclonal amplifications *of ERBB2, FGFR1/FGFR2,* and *JAK2* at early sampling time points, undetected in cfDNA, suggesting the presence of minor but relevant subpopulations that may contribute to resistance and represent emerging targets for therapeutic intervention. Both cfDNA and CTCs demonstrated loss of *BAP1* and *BRCA2* suggesting impaired DNA damage repair. As for the patient above, emerging evidence supports the use of PARPi olaparib. The clinical relevance of *BAP1* loss remains under investigation but its co-occurrence highlights broader genomic instability and potential vulnerability to DNA repair–targeted therapies [46, 47]. In addition, the *TP53* H179Q mutation highlights the current therapeutic gap in directly targeting mutant p53, although novel strategies including small molecules, gene therapy and immunotherapies are being actively explored [48, 49]. Finally, unique CTC-specific mutations (*FOXA1* V229I*, PIK3CA* N114D*, PTEN* S227F and *TP53* S215G) suggest ongoing real-time clonal evolution of the disease, further supporting the utility of parallel cfDNA and CTC analyses in capturing tumour heterogeneity.

For patient 3, the limited data preclude clinical interpretation. Nonetheless, it is notable that cfDNA failed to capture the genomic changes observed in the 2 CTCs analysed. Potentially these CTCs represent aggressive subclones that are minimally detected in plasma cfDNA due to their low representation or persistence as viable, non-apoptotic cells.

Throughout patient 4’s disease, *PIK3CA* H1047R and *TP53* G245V driver mutations persisted in cfDNA and CTCs. As previously mentioned, clinical trials indicate the use of fulvestrant and alpelesib [7], alpelisib plus nab-paclitaxel in HER2- MBC [44] or alpelisib + sacituzumab govetican (NCT05143229). The detection of amplification of the region containing *FGFR2* in multiple CTCs that was undetected in all cfDNA samples highlights the ability of CTC profiling to capture subclonal or site-specific alterations that may be missed in plasma cfDNA. The ReFocus trial (NCT04526106), a phase 1/2 open label global study, is currently evaluating the safety and efficacy of RLY-4008 in patients with advanced unresectable and/or metastatic cancers harbouring an *FGFR2* alteration [50]. Additionally, further investigations are ongoing to assess the efficacy of FGFR inhibitors such as futibatinib, erdafitinib and pemigatinib to target *FGFR2* alteration in BC [51].

Patient 5 showed progression at all time points, with no response to treatment changes. CTCs indicated resistance to CDK4/6 inhibitor (through loss of the region containing *RB1*) at the earliest time point whilst the patient was receiving palbociclib, that was not detected in the cfDNA. Mutation analysis revealed *PIK3CA M1043V* in all cfDNA samples plus an individual CTC (only 2 tested), suggesting the addition of alpelesib to fulvestrant at the earliest sampling point. Furthermore, sWGS analysis revealed gain of the region containing *ERBB2* in cfDNA and CTCs, indicating either a switch from HER2-negative metastatic disease or the expansion of a pre-existing subclonal HER2-amplified population, potentially responsible for lack of response to lines of treatment. Targeted intervention against HER2 could have meaningfully altered the patient’s treatment trajectory. To date there is no routine screening to monitor for such change, which can significantly impact therapeutic management [52]. Amplification of the region containing *KRAS* was also concurrently observed, activating the RAS/MAPK pathway and potentially diminishing the effectiveness of HER2-targeted therapies. In this case, combining anti-HER therapy with MEK inhibitors could overcome resistance by inhibiting parallel oncogenic signalling pathways [53]. Amplification of the *FGFR1* region was detected post-AI treatment, consistent with FGFR alterations commonly seen in endocrine-resistant patients [54, 55]. Screening for *FGFR1* gene amplification might represent a viable strategy to identify patients eligible for treatment by FGFR inhibition [56]. Results of the RADICAL trial (NCT01791985) demonstrated that addition of FGFR inhibitor AZD4547 to AI treatment in patients who have become resistant to this treatment can be beneficial in a subset of patients [57]. In addition, research is currently underway investigating the potential of pan-TKI to target FGFR1-4 alterations [58].

Patient 6, also had amplification of the region containing *ERBB2* detected in cfDNA and CTCs pointing towards the use of targeted anti-HER therapy to slow disease progression. As with patient 5, concurrent amplification of the region containing *KRAS* was observed, suggesting potential benefit from a combination of anti-HER2 therapy with MEK inhibitors [53]. Coupled with untreated HER2 pathway activation, loss of the region containing the *KDM6A* gene would also fuel drug resistance [59].

For patient 7, both cfDNA and CTCs revealed amplification of the region containing PIK3CA at the earliest sampling point, coinciding with disease progression on eribulin, supporting the rationale for considering combination therapy with a PI3K pathway inhibitor. Lastly, patient 8 had amplification of the region containing *KRAS* detected in both cfDNA and CTCs, indicating that targeting the RAS/MAPK pathway (MEK inhibitors) could potentially slow disease progression.

### Limitation and technological challenges

Limitation of this study include the small patient cohort and relatively low success rate of single-CTC sequencing, with about half of CTCs failing to meet sWGS quality thresholds for successful analysis meaning that the analysis is inevitably dependent on the success of cell recovery and downstream sequencing. Single CTCs identified by CellSearch® were selected through visual inspection; the majority that were successfully sequenced had a cancer profile, which underscores the importance of rigorous morphological review and awareness of the documented high inter-reader agreement for CTC definition [60]. Notably, sCNA patterns were largely clonal, highlighting a second potential limitation of CellSearch®, which enriches for EpCAM expressing CTC populations, failing to capture non-EpCAM subpopulations and reinforcing the need for broader and more efficient platforms [61, 62]. Conversely, the richer genomic data obtained from CTC sWGS likely reflect both biological and technical factors. CTCs provide a pure tumour fraction with intact, non-fragmented DNA that enables more uniform coverage, while also capturing viable, treatment-resistant subclones that are underrepresented in cfDNA. In contrast, cfDNA reflects a stochastic mixture of fragmented DNA from both tumour and non-tumour cells, which can dilute or obscure low-frequency tumour derived DNA and limit the detection of certain CNAs and mutations. Serial genomic analysis of CTCs and cfDNA remains technically demanding due to low CTC numbers, sample heterogeneity and complex workflows. Standardized collection, optimized enrichment and improved single-cell sequencing methods will be essential to improve success rates. Importantly, WGA introduces bias, including allelic dropout and reduced SNV accuracy; eliminating WGA and applying amplicon-based hotspot analysis has been shown to improve coverage uniformity and mutation call reliability [63]. Ultimately, further studies on larger cohorts will be critical to advance CTC genomic profiling as part of a robust, multi-analyte strategy in the clinic.

## Conclusions

Overall, this study underscores the value of CTC profiling in identifying subclonal, treatment-resistant clones with metastatic potential at the time they first emerge. These cells represent a small but clinically significant population, distinct from the bulk tumour mass that contributes the majority of ctDNA to plasma. Notably, HER2 status changes were detected in both cfDNA and CTCs of 2 of 8 patients, suggesting the potential under-detection of receptor conversion in relapsed disease, such blood-based monitoring could be important for many patients. In addition, CTCs revealed copy number alterations and unique mutations not captured by cfDNA, reinforcing their role as a complementary biomarker. Conversely, some low-VAF ctDNA mutations were not detected in CTCs, reflecting tumour heterogeneity and emphasizing the advantage of a multi-analyte approach to capture the full landscape of evolving disease for guiding treatment.

## Supplementary Information

Supplementary Material 1

Supplementary Material 2

## Data Availability

Data available upon reasonable request to js39@leicester.ac.uk.

## Abbreviations

BC: Breast cancer
MBC: Metastatic breast cancer
cfDNA: Circulating free DNA
ctDNA: Circulating tumour DNA
CTC: Circulating tumour cell
HER2: Human epidermal growth factor receptor 2
ER: Estrogen receptor
SNV: Single nucleotide variant
CNA: Copy number alteration
sCNA: Somatic copy number alteration
cfDNA: Cell-free DNA
WGA: Whole genome amplification
K2 EDTA: Dipotassium ethylenediaminetetraacetic acid
FFPE: Formalin-fixed paraffin-embedded
sWGS: Shallow whole genome sequencing
QC: Quality control
MAPD: Median absolute pairwise difference
TFx: Tumour fraction
CN: Copy number
HETD: Hemizygous deletion
NEUT: Copy neutral
GAIN: Copy gain
AMP: Amplification
HLAMP: High-level amplification
WBC: White blood cell
ALP: Alkaline phosphatase
CHIP: Clonal haematopoiesis of indeterminate potential
SNP: Single nucleotide polymorphism
VAF: Variant allele frequency
IGV: Integrative genomics viewer
