# Supplementary material for: Concurrent genomic assessment of circulating tumour cells and ctDNA to guide therapy in metastatic breast cancer
Source: BMC Cancer. 2025 Dec 5;25:1858. doi: 10.1186/s12885-025-15187-5 (PMC12681140; doi:10.1186/s12885-025-15187-5)
Supplement: Supplementary file 2 — Supplementary Material 2 [file 12885_2025_15187_MOESM2_ESM.pdf]

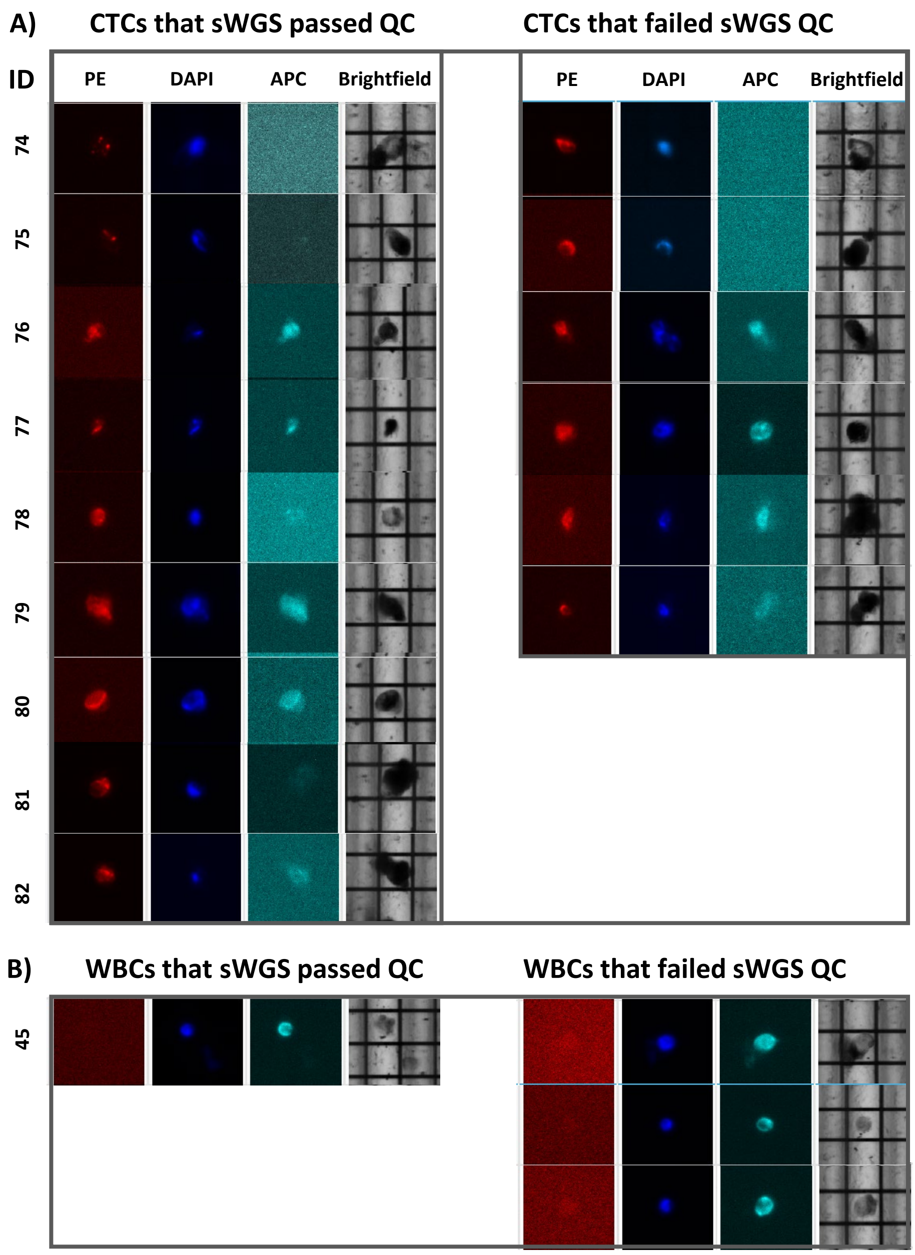


**Supplementary Figure 1.** Representative CellSearch®-defined single cells from Patient 5, including **A)** CTCs and **B)** WBCs, recovered by DEPArray. All cells were visually selected for sWGS; those on the left achieved the sWGS QC threshold, whereas those on the right failed to meet QC.

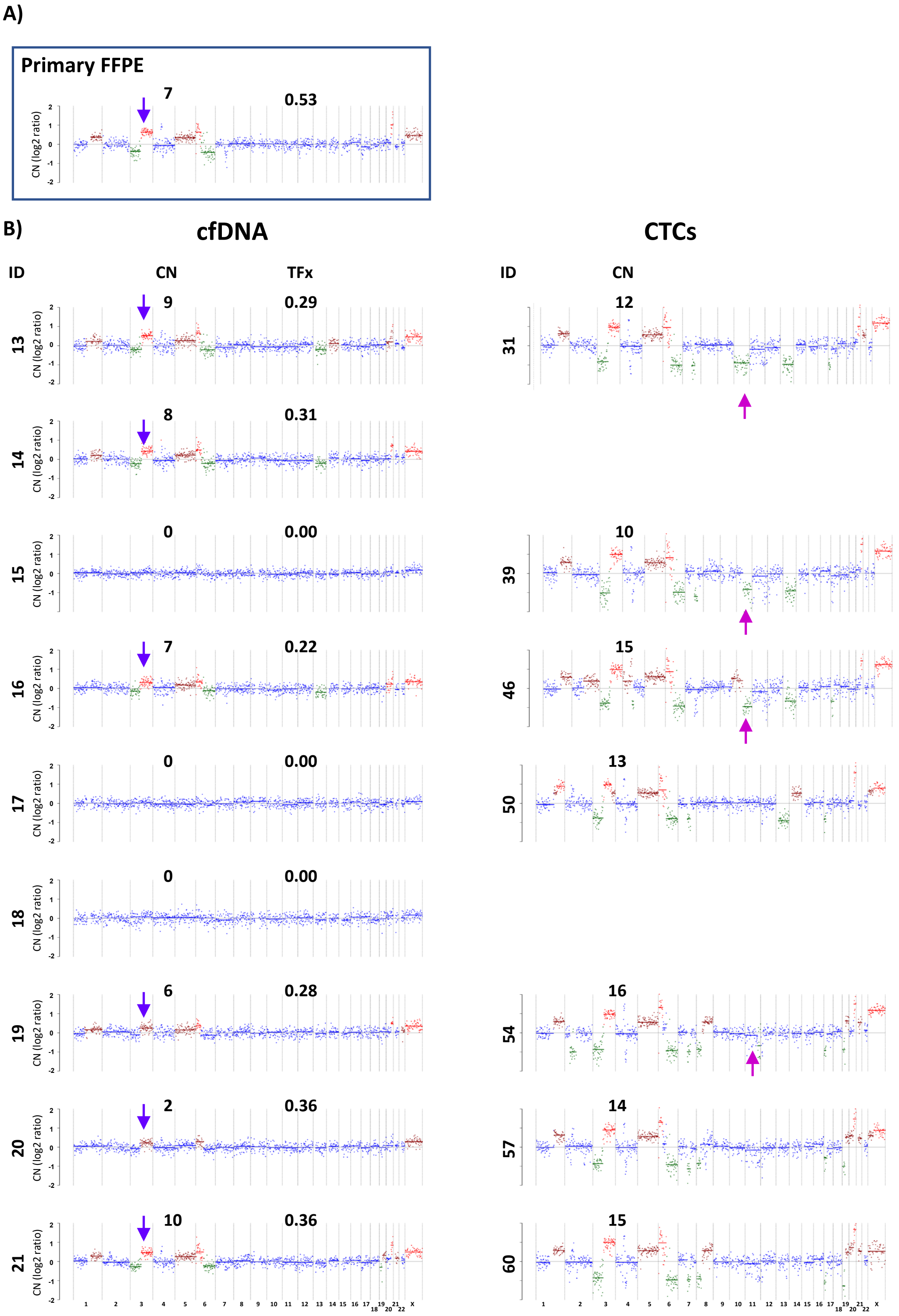


**Supplementary Figure 2.** Patient 2 sWGS-sCNA profiles of **A)** primary disease FFPE and **B)** MBC cfDNA and single CTCs showing gain of the region containing *PIK3CA* (purple arrow) and loss of the region containing *PTEN* (magenta arrow).

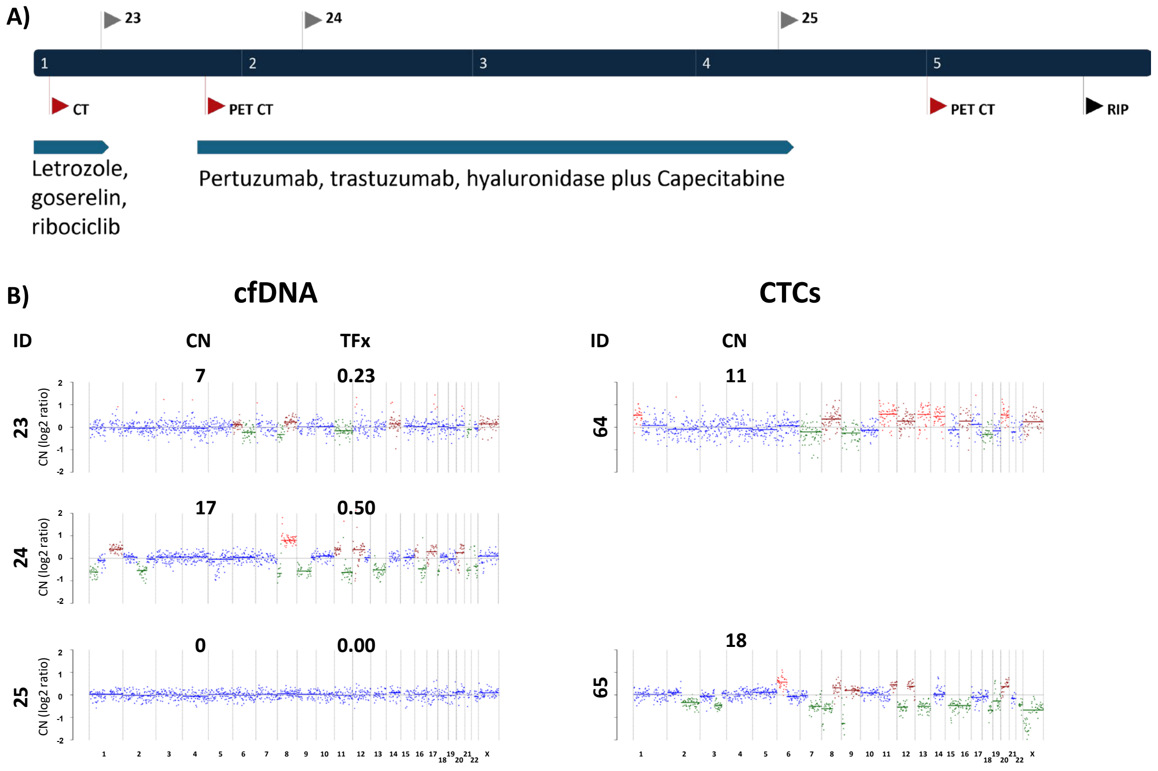


**Supplementary Figure 3. Patient 3 sWGS-sCNA analysis. A)** Treatment and sampling timeline whereby sample ID is highlighted above the timeline and red flags below the timeline indicate radiologically confirmed disease progression. **B)** sWGS-sCNA profiles of cfDNA and single representative CTCs emphasising the ultra-rich data from single-cell sequencing that can be missed in matched cfDNA alone.

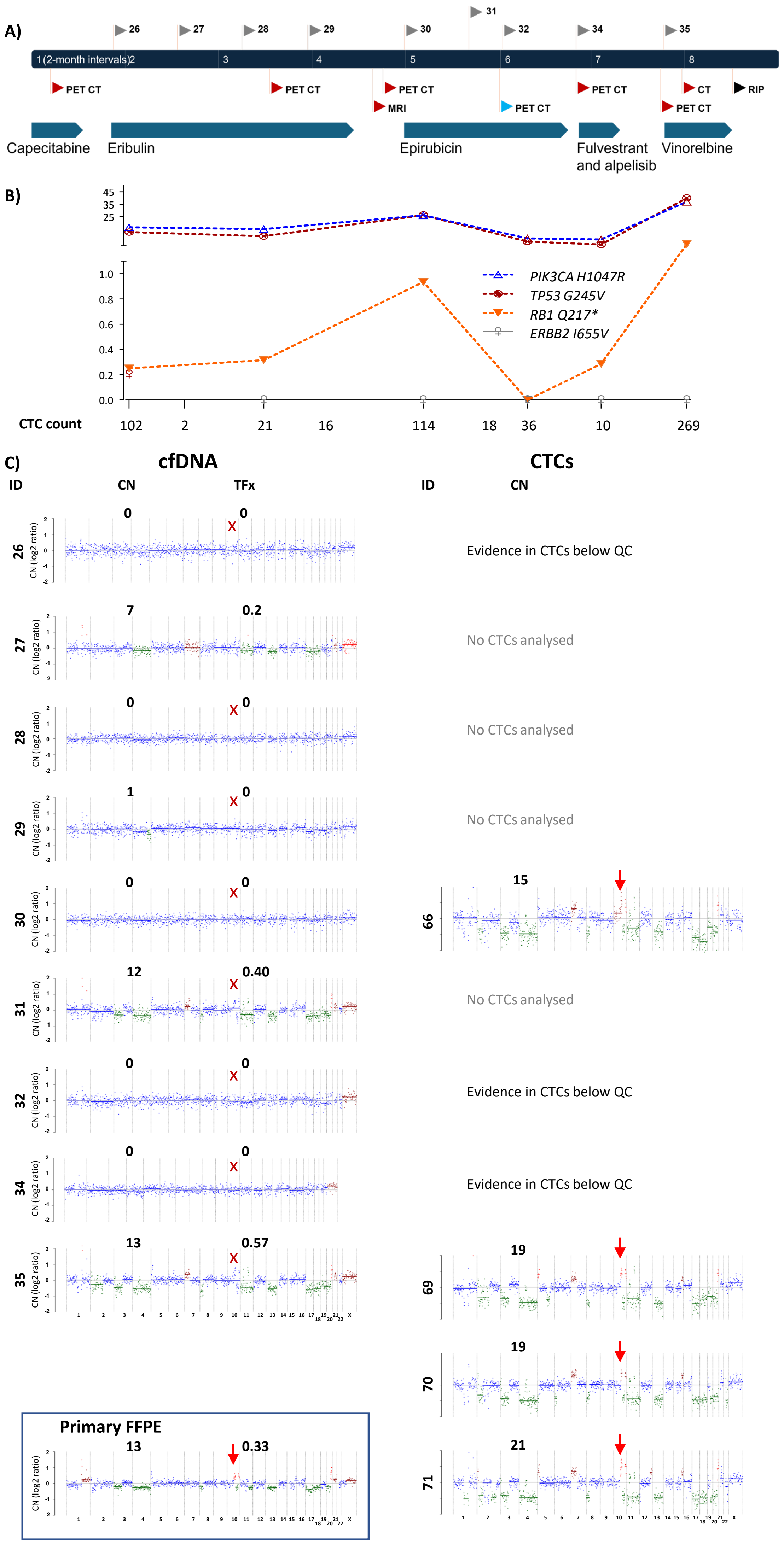


**Supplementary figure 4. Patient 4 longitudinal sWGS-sCNA and mutation analysis in CTCs and cfDNA. A)** Treatment and radiological response timeline, with cfDNA sample IDs shown above the timeline. Blue and red flags below indicate stable disease and disease progression, respectively. **B)** Mutation analysis of cfDNA (samples 27, 28, 29, 31, 33, 34 and 35) with CTC counts at each time point on the x-axis. The dotted line indicates that the mutation was concurrently detected in CTCs. **C)** sWGS copy number profiles, showing amplification above the normal (blue) baseline of the region of chromosome 8 containing *FGFR1* (red arrows) also evident in primary tissue FFPE.

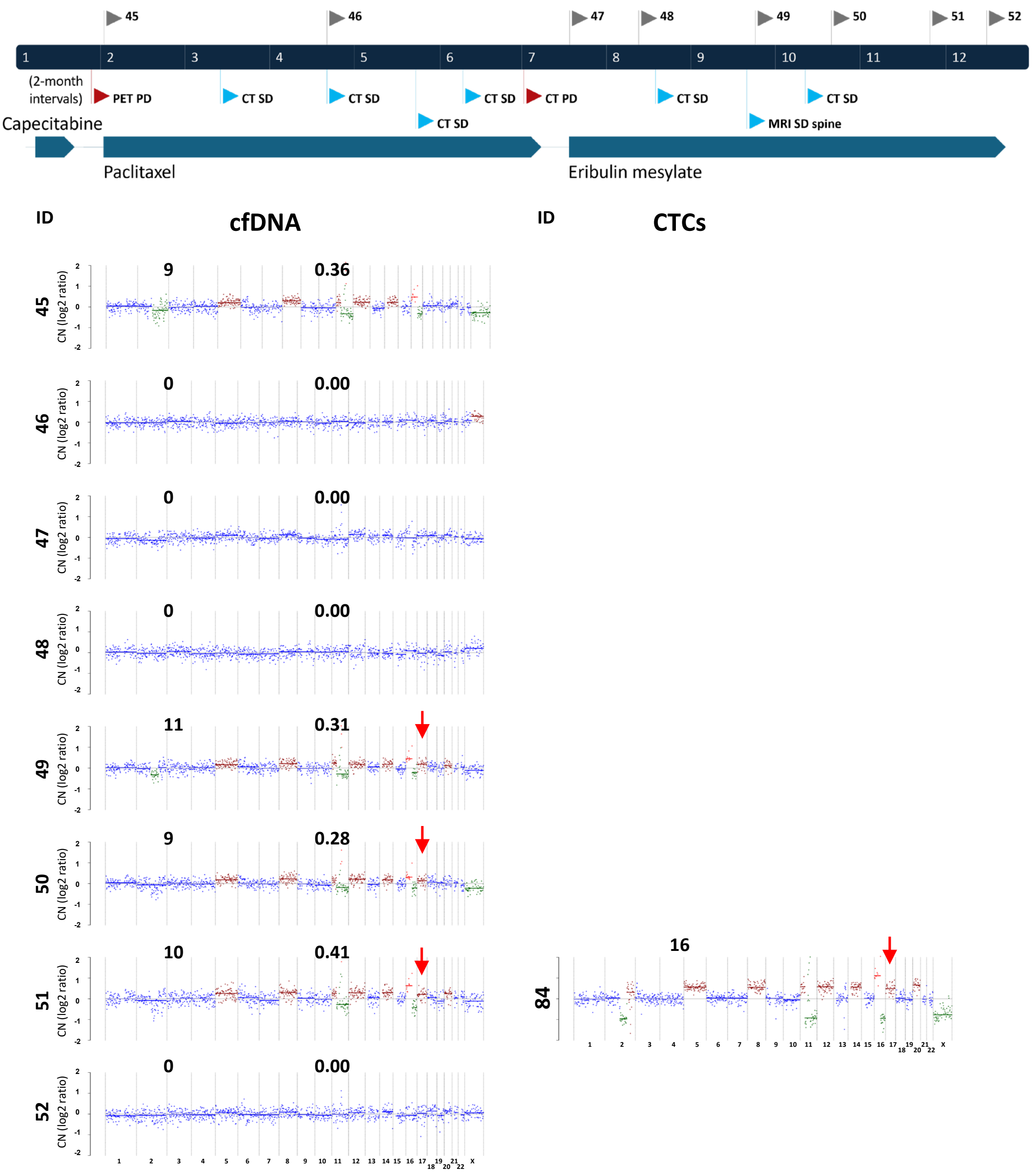


**Supplementary Figure 5. Patient 6 sWGS-sCNA analysis. A)** Treatment and radiological response timeline, with cfDNA sample IDs shown above the timeline. Blue and red flags below indicate stable disease and disease progression, respectively. sWGS indicates a switch from HER2-negative primary disease, with amplification of the region containing *ERBB2* (red arrow) in sequential cfDNA samples plus a representative CTC.

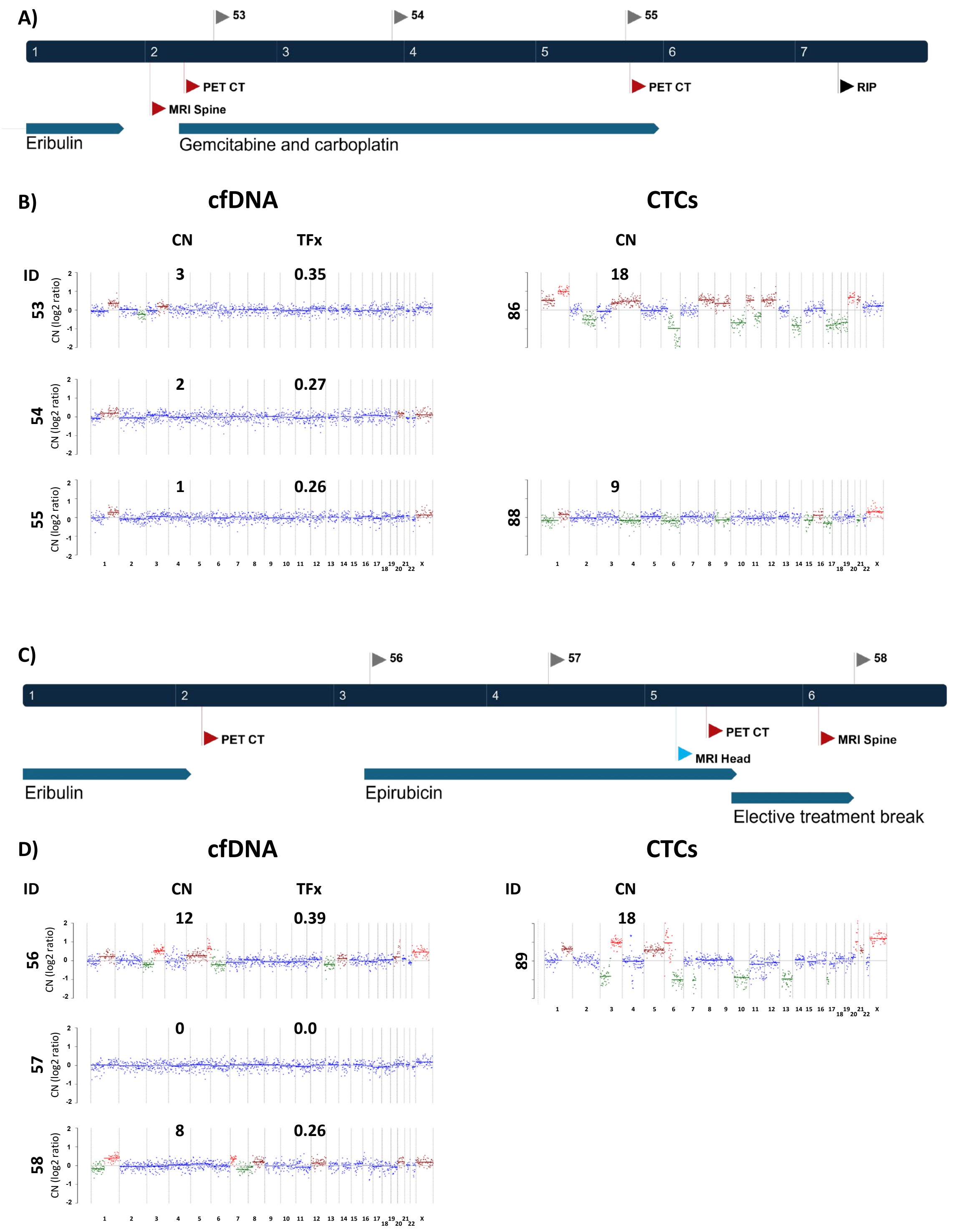


**Supplementary Figure 6. A) Patient 7** treatment and sampling interval timeline whereby sample ID is highlighted above the timeline and red flags below the timeline indicate radiologically confirmed disease progression. **B)** sWGS-sCNA profiles of cfDNA and single representative CTCs. **C) Patient 8** treatment and sampling interval timeline. **D)** sWGS-sCNA profiles of cfDNA and single representative CTCs.
